# Anti-cancer potential of persimmon (*Diospyros kaki*) leaves via the PDGFR-Rac-JNK pathway

**DOI:** 10.1038/s41598-020-75140-3

**Published:** 2020-10-22

**Authors:** Heon-Su Kim, Jung-Soo Suh, Yoon-Kwan Jang, Sang-Hyun Ahn, Ganesan Raja, Jin-Chul Kim, Youngmi Jung, Sang Hoon Jung, Tae-Jin Kim

**Affiliations:** 1 Department of Integrated Biological Science, Pusan National University, Pusan, 46241 Republic of Korea; 2 Department of Biological Sciences, Pusan National University, Pusan, 46241 Republic of Korea; 3 Natural Product Informatics Research Center, Korea Institute of Science and Technology (KIST), Gangneung, 25451 Republic of Korea; 4 Institute of Systems Biology, Pusan National University, Pusan, 46241 Republic of Korea; 5 Natural Product Research Center, Korea Institute of Science and Technology (KIST), Gangneung, 25451 Republic of Korea

**Keywords:** Biochemistry, Biological techniques, Cancer, Cell biology, Drug discovery

## Abstract

Persimmon leaves are known to have some beneficial effects, including ROS elimination, lipid circulation, and neuronal protection. However, their anti-cancer properties and the underlying mechanisms remain unclear. Herein, we show that treatment with the ethanol extract of persimmon, *Diospyros kaki,* leaves (EEDK) induces cancer cell death and inhibits cell proliferation. Using fluorescence resonance energy transfer (FRET) technology with genetically-encoded biosensors, we first found that EEDK stimulates a PDGFR-Rac signaling cascade in live cells. Moreover, we found that downstream of the PDGFR-Rac pathway, JNKs are activated by EEDK. In contrast, JNK-downstream inhibitors, such as CoCl2, T-5224, and pepstatin A, attenuated EEDK-induced cell death. Thus, we illustrate that the PDGFR-Rac-JNK signaling axis is triggered by EEDK, leading to cancer cell death, suggesting the extract of persimmon leaves may be a promising anti-cancer agent.

## Introduction

Cancer is a significant health problem, and one of the deadliest diseases globally. In order to cure a variety of cancers, researchers have suggested diverse treatment methods such as immunotherapy, gene therapy, and nanomedicine. Chemotherapy, known as a common type of cancer treatment along with surgery and radiotherapy, has been widely used in modern cancer treatment as a curative option^1,2^. The advance of molecular biology and genetics has enabled “targeted chemotherapy” to treat tumors with drugs targeting abnormal molecules that are only in cancer cells^3^. However, even precisely designed anti-cancer drugs can damage healthy cells in addition to cancer cells, mainly because they are cytotoxic. Natural products have been considered as alternative anti-cancer agents because they do not induce unwanted cytotoxic effects in the same way as synthetic drugs^4–6^. Compounds derived from edible natural products are more efficacious; since many substances produce synergy, natural compounds have better effects even with a relatively small amount than a single purified component^7,8^, and cause fewer side effects; they are relatively less virulent for healthy cells compared to synthetic drugs. Furthermore, many natural products suppress cancer cell growth through antioxidant and anti-mutagenic activities because they contain various bioactive compounds, such as alkaloids, polyphenols, and flavonoids^9,10^. Therefore, we must investigate these novel beneficial natural products and evaluate their potential as novel anti-cancer treatments.

Persimmon (*Diospyros kaki* Thunb.) belongs to the Ebenaceae family of plants and is generally cultivated throughout Korea, China, Japan, and East Asia. While its fruits are widely consumed as food, the leaves of *D. kaki* are often used as a herbal tea and in traditional medicine because of their beneficial properties^11^. *D. kaki* leaves are rich in bioactive compounds, including, but not limited to, polyphenol, flavonoids, and terpenoids^12^. Ethanol extracts of *D. kaki* leaves (EEDK) contain quercetin and kaempferol and its glycoside, galactoside, or galloylated derivatives^13^. EEDK plays essential roles in radical-scavenging and inhibition of inflammatory mediators and has anti-oxidative activity^14–16^. Moreover, recent studies have shown that EEDK ameliorates neurodegeneration and ocular hypertension^17,18^. While the pharmacological mechanisms of EEDK have been well studied and understood, the anti-cancer effects of EEDK and their underlying mechanisms remain unclear.

The aim of the present work was, thus, to verify the anti-cancer efficacy of EEDK and reveal the underlying mechanism(s) along with any potential novel biochemical pathways. We adapted fluorescent resonance energy transfer (FRET)-based biosensors to visualize EEDK-mediated cell signaling behavior at the single-cell level. Our findings demonstrate that EEDK stimulates anti-cancer activity through the PDGFR-Rac-JNK signaling cascade.

## Results

### The effects of EEDK on cell viability and colony formation in HepG2 and HEK293A cells

**Figure 1:**
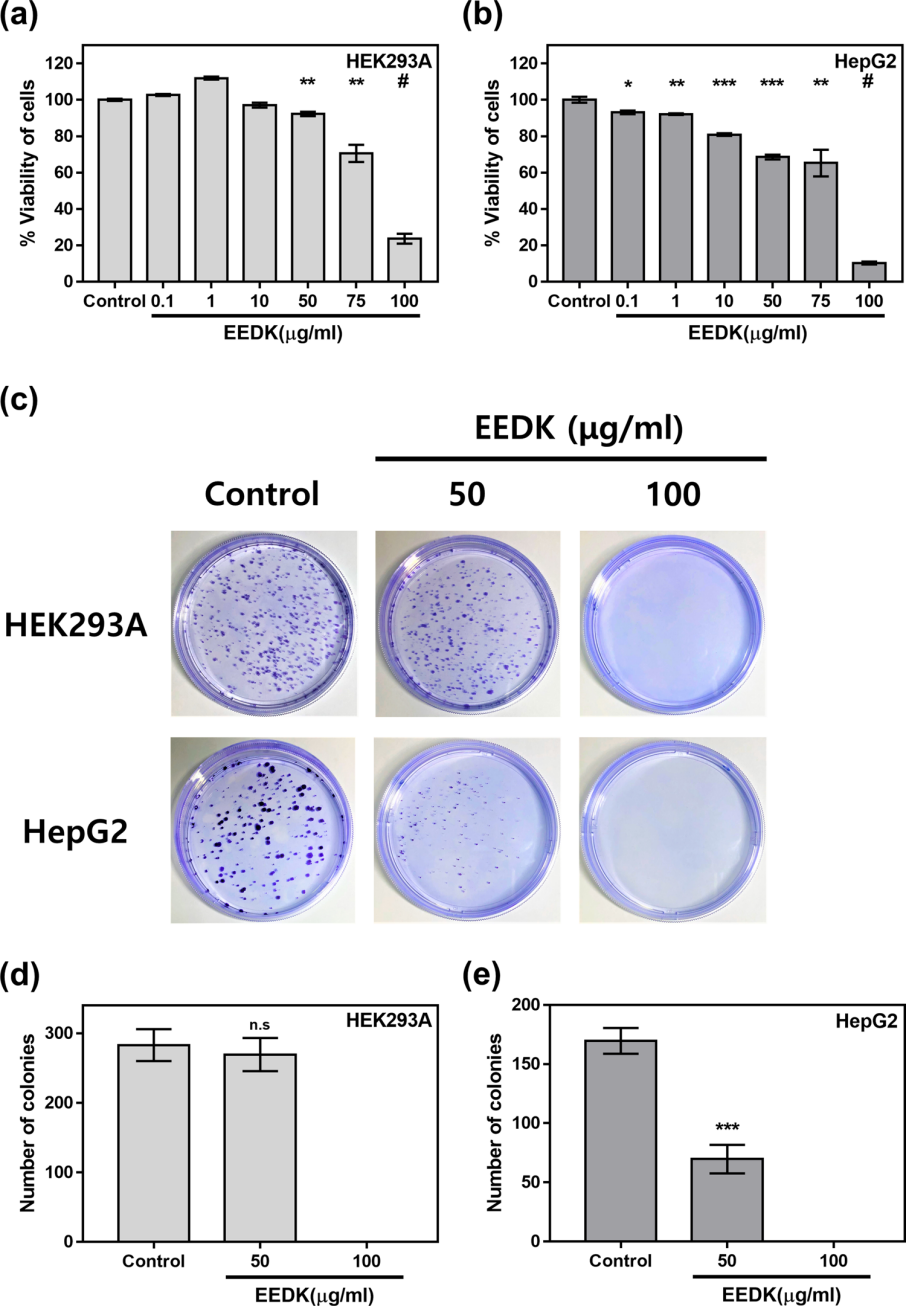
The effects of EEDK on cell death and proliferation. (**a**,**b**) viability of (**a**) HEK293A and (**b**) HepG2 cells exposed to control (0.5% (v/v) DMSO) and EEDK (0.1 to 100 μg/mL) for 24 h, as measured using viability assays. The bar graphs describe mean values of cell viability with error bars indicating the Standard error of the mean(S.E.M) (n = 3, *P < 0.05, **P < 0.01, ***P < 0.001, and ^#^*P* < 0.0001, Student t-test). Absorbance values of solubilized formazan product were measured using the Glomax Multi + Microplate Multi Reader (9301-010. Promega, USA). (**c**) Clonogenic assay images of HEK293A and HepG2 cells exposed to control (0.5% (v/v) DMSO) and EEDK (50 and 100 μg/mL) for 30 h. The medium was replaced with growth medium. (**d**,**e**) The bar graphs represent the mean number of colonies, with error bars indicating the S.E.M (n = 3, n.s > 0.05, ***P < 0.001, student t-test). Colony pixel is > 20, circularity is 0.2–1.0. Quantification of cell colonies was performed using the ImageJ software.

To assess the cytotoxic effects of EEDK on HEK293A (normal cell) and HepG2 (cancer cell) lines, cells were treated with EEDK, and cell viability was analyzed using the WST-8 assay. We found that EEDK exerts cytotoxic effects on both cell lines in a dose-dependent manner (Fig. 1). In HEK293A cells, significant cytotoxic effects were observed at high concentrations of EEDK (50–100 μg/mL) but not at low concentrations (0.1–10 μg/mL; Fig. 1a). The survival rate of HepG2 cells decreased in a dose-dependent manner, even at low concentrations of EEDK (0.1–1 μg/mL; Fig. 1b). Next, a clonogenic assay was performed to determine the effect of EEDK on cell proliferation and colony formation (Fig. 1c–e). The number of colonies decreased significantly in HepG2 cells treated with 50 μg/mL EEDK (Fig. 1e). This was not the case with the HEK293A cell line (Fig. 1d). No visible colonies were observed in both cell lines at 100 μg/mL EEDK. These data suggest that EEDK suppresses cancer cell survival and colony formation, making it a potentially promising anti-cancer agent.

### EEDK increases intracellular Ca^2+^ concentration and βTrCP expression

**Figure 2:**
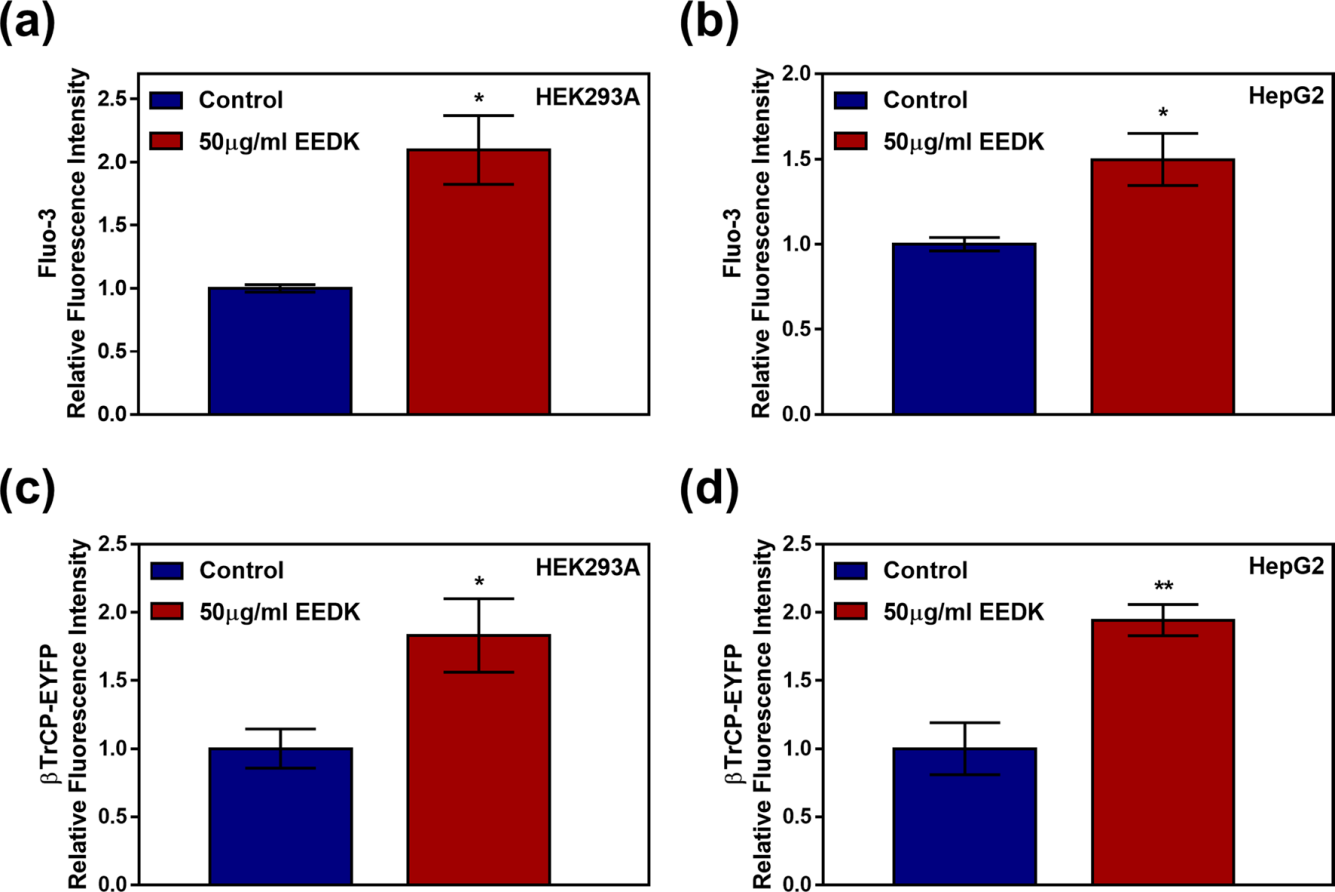
The effects of EEDK on intracellular Ca^2+^ concentration and βTrCP-EYFP expression. (**a**,**b**) The intracellular Ca^2+^ concentration of (**a**) HEK293A and (**b**) HepG2 cells exposed to control (0.5% (v/v) DMSO) and EEDK (50 μg/mL) for 8 h, as measured using the Fluo-3 assay (n = 3). (**c**,**d**) βTrCP-EYFP expression in (**c**) HEK293A and (**d**) HepG2 cells exposed to control (0.5% (v/v) DMSO) and EEDK (50 μg/mL) for 24 h (n = 4). Fluorescence values of the Ca^2+^-Fluo-3 complex and expressed βTrCP-EYFP were detected using the Glomax Multi + Microplate Multi Reader (9301–010; Promega, USA). The bar graphs describe mean values of relative fluorescence, with error bars indicating the S.E.M (*P < 0.05, **P < 0.01, Student’s *t*-test).

EEDK has previously been reported to inhibit Wnt/β-catenin signaling activity. Moreover, EEDK inhibition has been shown to downregulate cyclin D1 mRNA expression^19^. However, the mechanism underlying the downregulation of Wnt/β-catenin signaling by EEDK is not fully understood. Since calcium signaling is a known negative regulator of Wnt/β-catenin signaling pathway^20^, we first investigated the role of EEDK in intracellular calcium signaling. As such, the Fluo-3 assay was performed to study the effect of EEDK on intracellular Ca^2+^ concentrations. Our data clearly show that EEDK treatment led to an increase in intracellular Ca^2+^ level in both cell lines (Fig. 2a,b), suggesting that EEDK may perturb the canonical Wnt/β-catenin signaling pathway by upregulating intracellular Ca^2+^ concentrations. We also considered that β-Transducin repeat-containing protein(βTrCP), an F-box/WD repeat-containing protein 1A, which is involved in β-catenin ubiquitination, may be another candidate inhibitor of Wnt/β-catenin signaling^21,22^. Thus, we designed a βTrCP- Enhanced yellow fluorescent protein(EYFP) plasmid which would allow us to quantify βTrCP expression by measuring EYFP fluorescence intensity. Interestingly, increased expression of βTrCP-EYFP was observed in EEDK-treated cells (Fig. 2c,d), suggesting that EEDK may downregulate the Wnt/β-catenin signaling pathway by activating βTrCP expression.

### The PDGFR pathway is involved in EEDK-induced cell death

**Figure 3:**
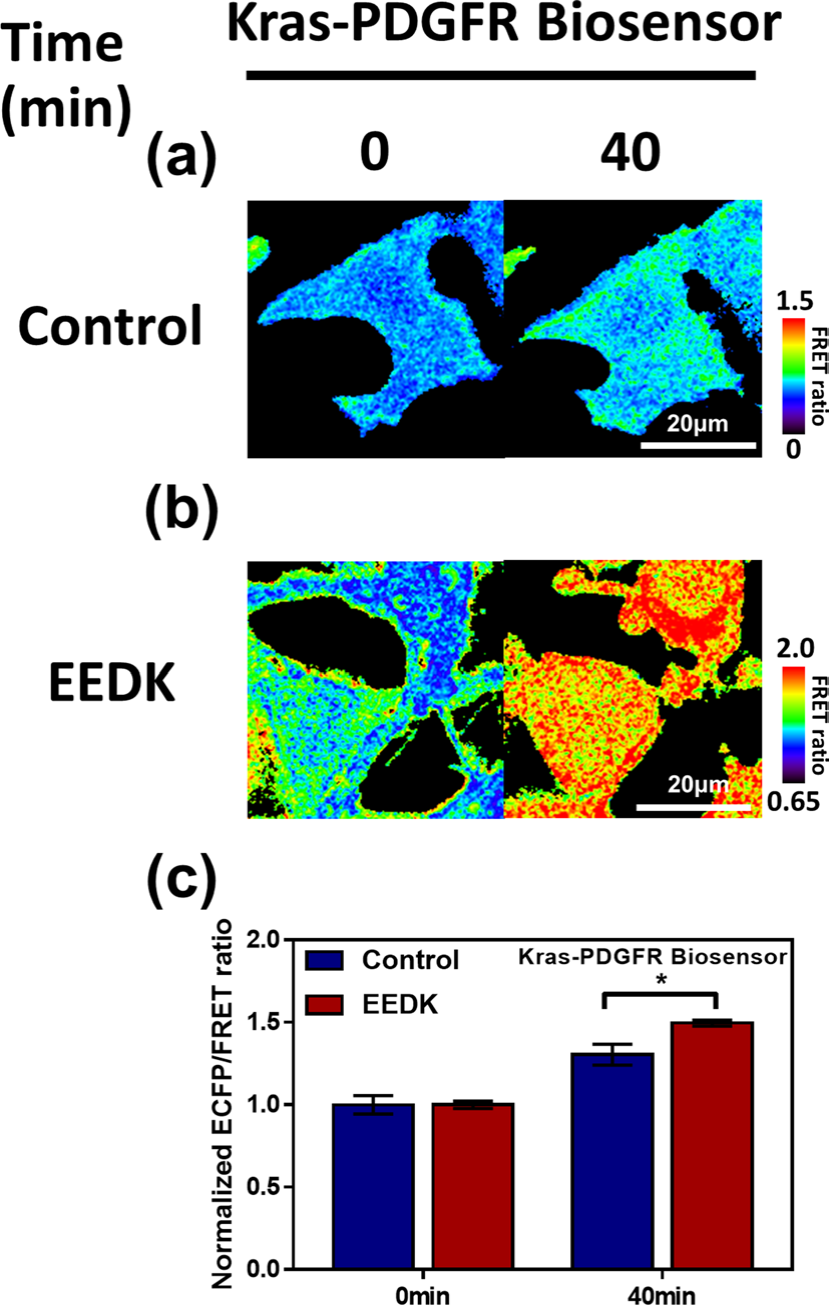
The effects of EEDK on PDGFR activity. (**a**,**b**) Time-lapse FRET images of the Kras-PDGFR biosensor in HEK293A cells exposed to the control (0.5% (v/v) DMSO) and EEDK (50 μg/mL). The colored scale bars represent the range of the ECFP/FRET emission ratios of the biosensors. Hot and cold colors indicate high and low PDGFR activity, respectively. Scale bar = 20 μm. (**c**) The bar graph describes the mean values of the normalized ECFP/FRET emission ratios of the biosensor at 0 min and 40 min. It contains error bars indicating the S.E.M (n = 7, *P < 0.05, Student’s *t*-test).

Platelet-derived growth factor receptors (PDGFRs), which are affiliated to class III receptor tyrosine kinases (RTKs), cooperate with integrin and contain many phosphorylated binding sites for adaptor proteins, like tyrosine-protein kinase Src(Src) kinase, Growth factor receptor-bound protein2(Grb2), Src homology region 2-containing protein tyrosine phosphatase 2(SHP2), and Non-catalytic region of tyrosine kinase(Nck)^23–25^. Accordingly, PDGFRs activate various downstream pathways (e.g. Ras-Microtubule associated protein kinase(MAPK), Phosphoinositide 3-kinase(PI3K)/Akt, Phospholipase Cγ(PLCγ), and c-Jun N-terminal kinase(JNK)-Stress-activated protein kinases(SAPK)) and play essential roles in cell proliferation, migration, differentiation, and survival^23^. To understand the role of EEDK in the activation of the PDGFR pathway, we utilized FRET technology along with a Kirsten rat sarcoma 2 viral oncogene homolog(Kras)-PDGFR biosensor^26^. The Kras-PDGFR biosensor was engineered to detect Tyr751 phosphorylation in the plasma membrane, the auto-phosphorylation site of PDGFR. When Tyr751 is phosphorylated, the conformational change induced in the biosensor results in an increased Enhanced cyan fluorescent protein(ECFP)/FRET emission ratio. Thus, the biosensor enabled us to directly visualize PDGFR activation upon EEDK treatment. The cells exposed to EEDK showed a substantial ECFP/FRET ratio change compared to the control-treated cells (Fig. 3); furthermore, the enhanced ratios between the two groups were significantly different (Fig. 3c). These data suggest that EEDK regulates cell survival by directly stimulating PDGFRs in the plasma membrane.

### EEDK plays a role in Rac activation

**Figure 4:**
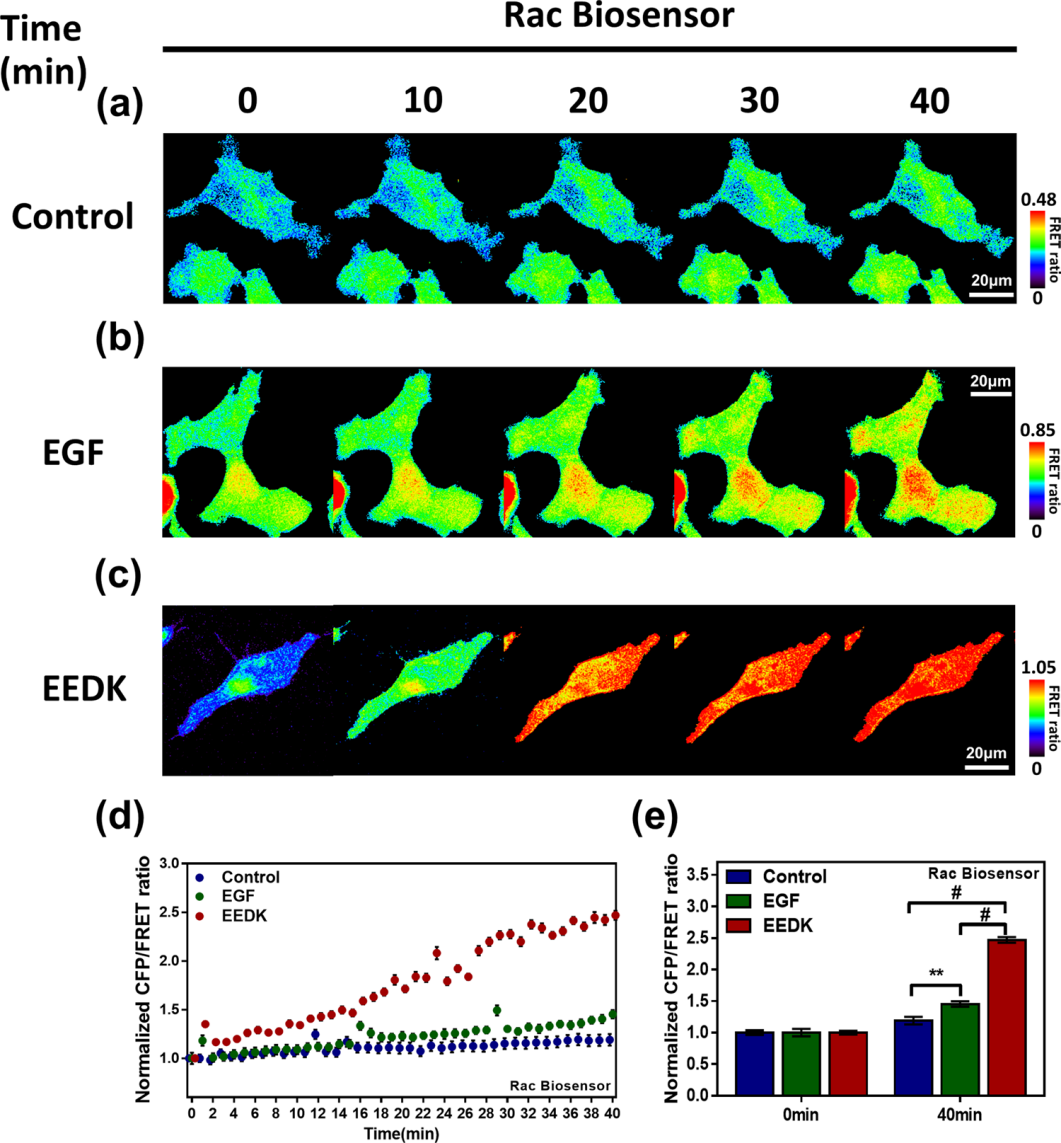
The effects of EEDK on Rac activity. (**a**–**c**) Time-lapse FRET images of the Rac biosensor in HEK293A cells exposed to control (0.5% (v/v) DMSO), EGF (100 ng/mL), and EEDK (50 μg/mL). EGF was used as a positive control. The color scale bars represent the range of the CFP/FRET emission ratio in the biosensors. Hot and cold colors indicate high and low Rac activity, respectively. Scale bar = 20 μm. (**d**) The time courses represent the average of the normalized CFP/FRET emission ratio changes of the Rac biosensor. The dots are mean values of normalized emission ratios, with error bars indicating the S.E.M (n = 8). (**e**) The bar graph describes the mean values of normalized CFP/FRET emission ratios of the biosensor at 0 min and 40 min. It also contains error bars indicating the S.E.M (n = 8, **P < 0.01, ^#^*P* < 0.0001, Student’s *t*-test).

Subsequently, we were curious to identify the effectors that were operated by PDGFR signaling. Among the PDGFR downstream effectors, Ras-related C3 botulinum toxin substrate (Rac), which can induce cell survival or death, depending on the cellular circumstance, was thought to be a potential effector. As a small Guanosine triphosphatase(GTPase), Rac cooperates with various regulatory proteins, especially guanine nucleotide exchange factors (GEFs), which stimulate the release of Guanosine diphosphate(GDP), leading to the formation of the Rac-GTP complex, and thereby contributing to Rac activation^27,28^. Activated Rac is involved in cell motility, the formation of lamellipodia, and membrane ruffling^29,30^. Since Rac activity also mediates several kinase activities, such as those of p38/MAPK and JNKs, it also influences cell growth, survival, and apoptosis^31,32^. To visualize real-time Rac activity, we employed Raichu-CRIB, a Rac biosensor engineered by Dr. Itoh^33^. When the activated Rac-GTP complex bound to the P21(Rac1) activated kinase 1(PAK1) domain of the biosensor, the distance between Cyan fluorescent protein(CFP) and Yellow fluorescent protein(YFP) increased the CFP/FRET ratio. We also found that EEDK promoted Rac activity in a time-dependent manner (Fig. 4a–d). Notably, EEDK-induced FRET changes were more significant compared to the Epidermal growth factor(EGF)-induced FRET changes (i.e. the positive control; Fig. 4e). These results suggest that EEDK may play an essential role in Rac signal activation.

### What are the effects of EEDK on JNK activity?

Next, we studied the effect of EEDK on JNKs, known mediators of cell survival and death. JNKs act as apoptosis-inducing double-edged swords and have both pro- and antiapoptotic functions^34,35^. There are a variety of downstream substrates that are phosphorylated by JNKs, including c-Jun, Activating transcription factor-2(ATF-2), Smad4, p53, and Bcl2-associated agonist of cell death(BAD). Since the expression of these modulators is dependent on cell type, the effect of the JNK pathway can be distinguished from the cellular environment and origin of cell^34,36,37^. For this experiment, we took advantage of FRET imaging to monitor JNK activity in living cells in response to direct treatment with EEDK. Developed by Dr. Komatsu, the hybrid Bioluminescence resonance energy transfer(hyBRET)-JNK-EV biosensor is composed of an Forkhead-associate 1(FHA1) domain, a flexible EV linker, and a JNK-specific substrate, which localizes between the FRET donor Tq2GL, a kind of CFP, and the acceptor, Yellow fluorescent protein for energy transfer(YPet)^38^. The FHA1 domain detects JNK-specific substrate phosphorylation, resulting in a conformational change in the sensor, and a subsequent increase in the FRET/Tq2GL emission ratio and JNK activity.

**Figure 5:**
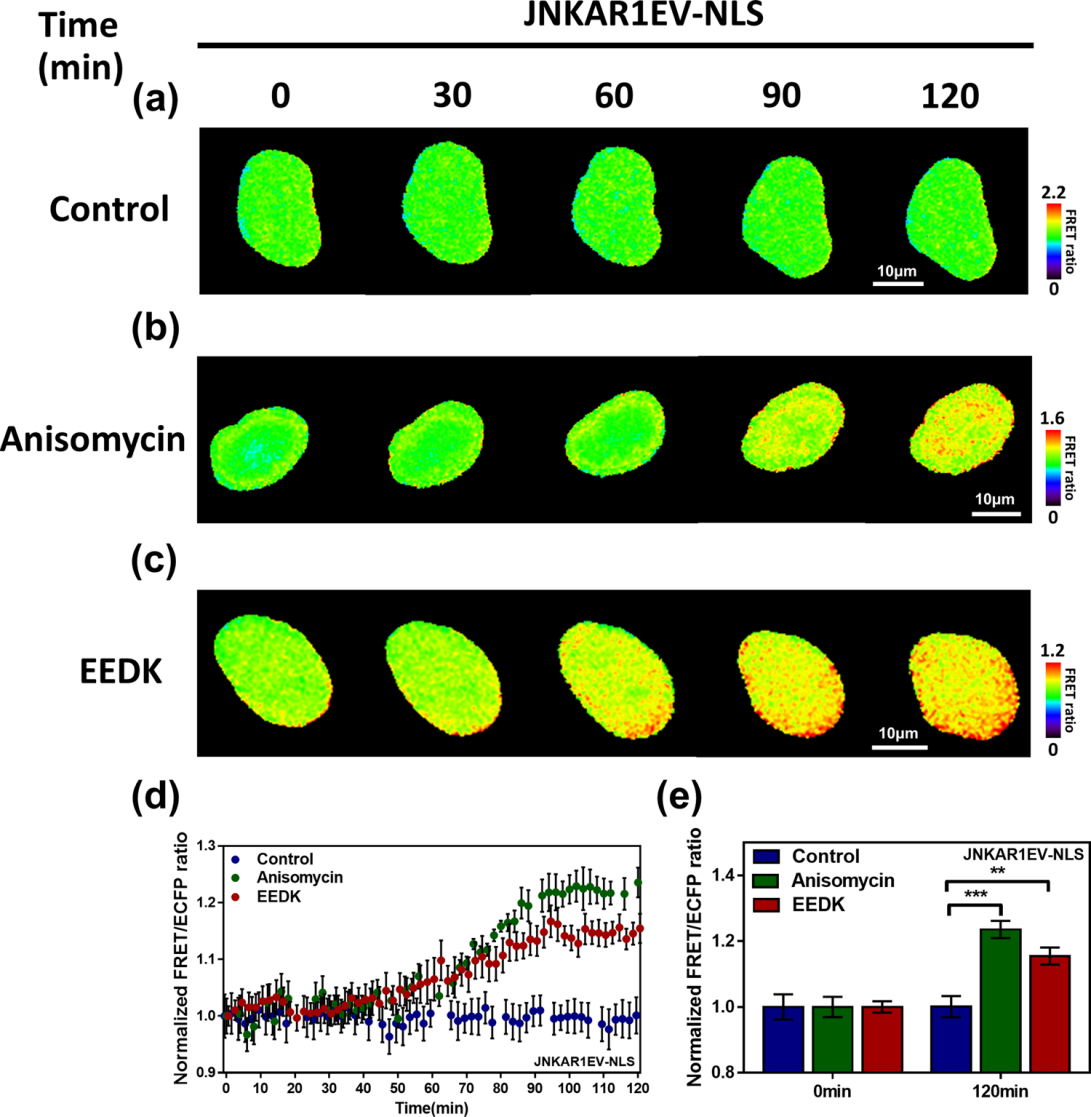
The effects of EEDK on the degree of JNK activation. (**a**–**c**) Time-lapse FRET images of JNKAR1EV-NLS in HEK293A cells exposed to the control (0.5% (v/v) DMSO), anisomycin (4 μM), and EEDK (50 μg/mL). Anisomycin was used as a positive control. The color scale bars represent the range of FRET/ECFP emission ratios of the biosensors. Hot and cold colors indicate high and low JNK activity, respectively. Scale bar = 10 μm. (**d**) The time courses represent the average of normalized FRET/ECFP emission ratio changes of JNKAR1EV-NLS. The dots are the mean values of normalized emission ratios, with error bars indicating the S.E.M (n = 6). (**e**) The bar graph describes the mean values of normalized FRET/ECFP emission ratios of the biosensor at 0 min and 120 min. The bar graph also contains error bars indicating the S.E.M (n = 6, **P < 0.01, ***P < 0.001, Student’s *t*-test).

It is important to investigate JNK activity in the cytosol and the nucleus. Once JNKs are activated by various upstream players, they translocate to the nucleus and phosphorylate a variety of transcription factors, including, but not limited to, c-Jun and ATF2^39,40^. Moreover, the JNK-specific substrate sequence used in the hyBRET-JNK-EV biosensor originated from the Jun dimerization protein 2(JDP2) protein, which is usually localized in nucleoplasm^41,42^. Hence, to visualize JNK activity in the nucleus, we developed a JNK biosensor attached to a nuclear localization sequence (NLS) using restriction enzyme cloning. This JNKAR1EV-NLS biosensor reveals an enhanced FRET/ECFP emission ratio when JNK activity is elevated. Following transfection with JNKAR1EV-NLS and starvation, cells were treated with dimethyl sulfoxide (DMSO), anisomycin, and EEDK (Fig. 5). As expected, DMSO treatment (i.e. the negative control) did not induce a change in the FRET ratio. However, anisomycin, a JNK activator, induced a conformational change in the biosensor, leading to an increase in the emission signal from YPet (Fig. 5a,b). In response to EEDK addition, the biosensor exhibited an enhanced FRET/ECFP ratio change (Fig. 5c,d). Furthermore, the increased ratio values between control and EEDK-treated cells were significantly different (Fig. 5e), suggesting that EEDK does indeed activate JNK activity.

### What are the effects of EEDK on the DNA-binding affinity of AP-1 and NFAT?

**Figure 6:**
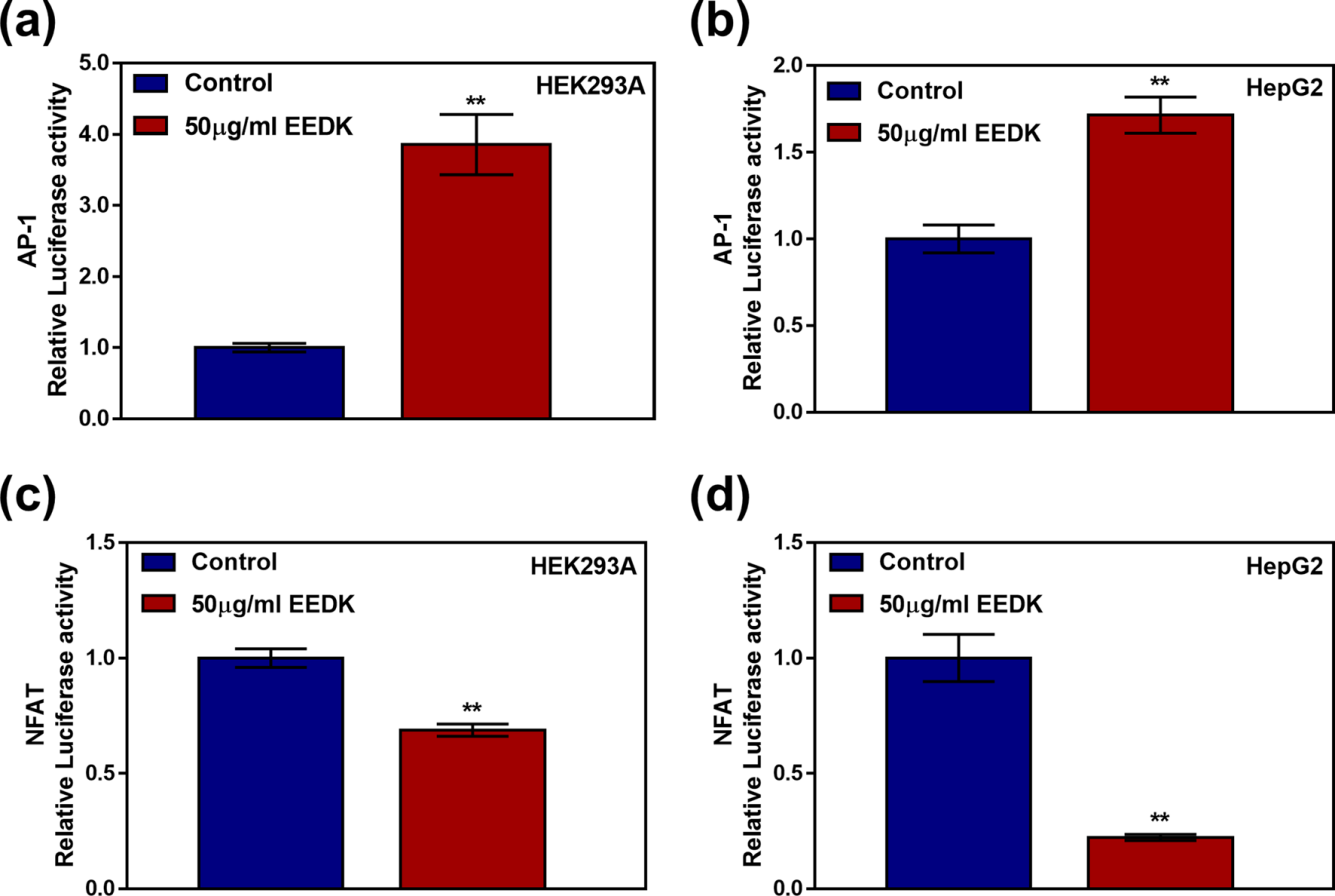
The effects of EEDK on the DNA binding activity of AP-1 and NFAT. (**a**,**b**) AP-1 activity of (**a**) HEK293A and (**b**) HepG2 cells exposed to the control (0.5% (v/v) DMSO) and EEDK (50 μg/mL) for 24 h. Cells were transfected with 3xAP1pGL3 (40342, Addgene). (**c**,**d**) NFAT activity of (**c**) HEK293A and (**d**) HepG2 cells exposed to the control (0.5% (v/v) DMSO) and EEDK (50 μg/mL) for 24 h. Cells were transfected with pGL3-NFAT luciferase (17870, Addgene). The bar graphs present mean values of relative luminescence values, with error bars indicating the S.E.M (n = 3, **P < 0.01, Student’s *t*-test). Luminescence values were detected using the Glomax Multi + Microplate Multi Reader (9301-010; Promega, USA).

Next, we were interested to study the mechanisms by which JNK activity induces cell death. Among the downstream candidates, Activator protein-1(AP-1) and Nuclear factor of activated T-cells(NFAT) were considered crucial transcriptional factors that are affected by JNKs. These transcriptional factors are essential for the regulation of the cellular immune system, and each one not only individually activates its own effector, but also cooperates to express various inflammatory genes, including, but not limited to, Interleukin-2(IL-2), Granulocyte–macrophage colony-stimulating factor(GM-CSF), and Interleukin-3(IL-3)^43^. To activate each transcription factor, however, AP-1 and NFAT require different cellular signaling cascades, the MAPK and calcineurin signaling cascades, respectively^44–47^. Since EEDK leads to a simultaneous increase in intracellular Ca^2+^ and activation of JNKs, we wondered how EEDK influences AP-1 and NFAT activity. We used a luciferase assay to reveal the effect of EEDK on AP-1 and NFAT activation. Consequently, the 3xAP1pGL3 and pGL3-NFAT plasmids, containing the DNA binding sites of AP-1 and NFAT, respectively, were transfected into both cell lines. We found that treatment with 50 μg/mL of EEDK resulted in increased AP-1 activity in both cell lines (Fig. 6a,b). These data suggest that EEDK-induces JNK phosphorylated c-Jun and upregulates AP-1 activity. However, the DNA-binding capacity of NFAT significantly decreased following treatment with EEDK (Fig. 6c,d). Previous studies have shown that the JNK signaling pathway leads to phosphorylation of NFAT to suppress its activity^48,49^. Therefore, our observations are in agreement with previous findings showing that EEDK activates the JNK pathway.

### JNK-downstream effector mediates EEDK-induced cell death

**Figure 7:**
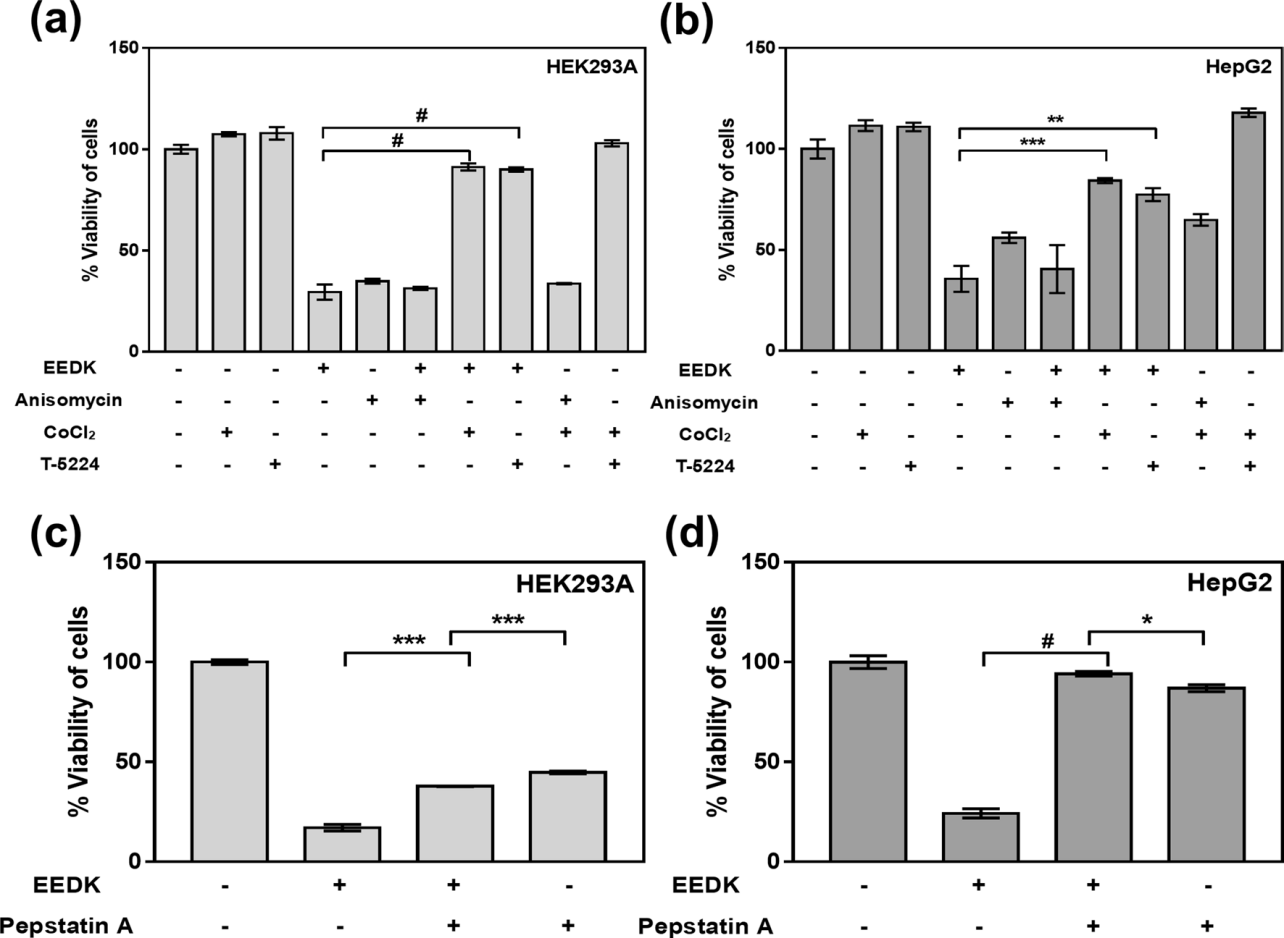
The effects of EEDK on cell death are dependent on JNK-AP-1/p53 activity. (**a**,**b**) Viability of (**a**) HEK293A and (**b**) HepG2 cells treated with or without the control (0.5% (v/v) DMSO), EEDK (100 μg/mL), anisomycin (JNK activator, 4 μM), CoCl_2_ (hypoxia mimicking agent, 150 μM), and T-5224 (AP-1 inhibitor, 10 μM) for 24 h, as measured using the viability assay (n = 4). (**c**,**d**) Viability of (c) HEK293A and (d) HepG2 cells treated with or without the control (0.5% (v/v) DMSO), EEDK (100 μg/mL), and pepstatin A (aspartic proteases inhibitor, 1 μM) for 24 h, as measured using the viability assay (n = 3). Anisomycin was used as a positive control. Pepstatin A is a known suppressor of p53 and TNF-α induced apoptosis. The bar graphs present the mean values of cell viability, with error bars indicating the S.E.M (*P < 0.05, **P < 0.01, ***P < 0.001 and ^#^*P* < 0.0001, Student’s *t*-test).

To confirm that the JNKs-AP-1 signal cascade leads to cellular death, several drugs associated with the JNK pathway were administrated (Fig. 7a,b). Anisomycin was used as the positive control for EEDK. As expected, anisomycin induced cell death in both cell lines. Next, we administrated T-5224, which inhibits AP-1 activity, with or without EEDK. Surprisingly, T-5224, an inhibitor of AP-1, significantly attenuated EEDK-induced cell death. It would seem that EEDK does not efficiently play a role in cellular death because of the suppressed DNA-binding capability of the AP-1 complex. The results showed that AP-1-induced cell death occurs following treatment with EEDK. Previous work has demonstrated that JNK activity and phosphorylation of c-Jun are diminished when cells are exposed to long-term (24–48 h) hypoxia^45^. To confirm that JNKs and p-Jun are activated by EEDK, we applied cobalt chloride(CoCl_2)_, a hypoxia mimicking agent, with EEDK.

Interestingly, the efficient application of CoCl_2_ reduced the rate of cell death. Thus, it can be inferred that a hypoxic environment successfully inhibits the JNK-AP-1 signaling cascade. The tendency of results was compatible between the two cell lines. These results showed that the addition of EEDK triggers the activation of the JNK-AP-1 pathway, resulting in cell death. We further analyzed pepstatin A, a known inhibitor of p53-induced apoptosis in lymphoid cells and Tumor necrosis factor-α(TNF-α) induced apoptosis in U937 cells^50,51^. Our data show that EEDK-induced cell death decreased following co-treatment with pepstatin A (Fig. 7c,d). Therefore, the downstream JNK cascades, including AP-1 and p53, are induced by high concentrations of EEDK, resulting in cell death.

## Discussion

Although chemotherapy has made a significant contribution to the prevention and treatment of cancer, unexpected side effects remain a considerable challenge^52^. Substances derived from natural products could be considered as potential alternatives. In particular, edible natural products are composed of bioactive compounds that cause fewer side effects^4–6^. Persimmon leaves have long been recognized as a traditional medicine and consumed in herbal teas due to their beneficial properties. Moreover, persimmon leaves are known to induce therapeutic effects, such as inhibition of oxidative stress, regulation of cholesterol accumulation, and improvement of blood circulation^15,17,53^. However, the putative anti-cancer effects of persimmon leaves and their mechanisms of action have, until now, remained unclear.

As illustrated in Fig. 1, viability assays revealed that HepG2 cells were more vulnerable to EEDK treatment than HEK293A cells. In addition, we performed a clonogenic assay, which showed that EEDK suppressed HepG2 cell proliferation, while HEK293A cells could still form colonies 30 h post-EEDK treatment. Why does the same EEDK concentration result in different outcomes depending on the characteristics of the cells? We suggest two potential theories to explain this conundrum. First, JNKs are the most crucial factor in EEDK-induced cell death. The JNK pathway is one of three well-known MAPK pathways, which mediate diverse cell signaling pathways^54^. Furthermore, there are many cell cascades (e.g. Signal transducer and activator of transcription 3(STAT3), Nuclear factor kappa-light-chain-enhancer of activated B cells(NF-κB), and p38) that correlate to JNKs^55–57^. Additionally, the degrees of activity of JNKs and their associated proteins differ significantly from cell to cell. The JNK family is composed of three members, JNK1, JNK2, and JNK3, with JNK1 and JNK2 being commonly expressed isoforms.

However, the roles of the three JNKs are poorly understood, and each JNK’s functions are different according to the cell type. For example, a series of studies have shown that JNK1 is responsible for cell survival, whereas JNK2 plays a critical role in cell death and apoptosis^58,59^. However, one study using mouse fibroblasts deficient in *Jnk1* or *Jnk2* founded that JNK1 is required for TNF-α-induced apoptosis, and that JNK2 contributes to the inhibition of apoptosis^60^. Additionally, JNKs are known as ‘Janus face,’ because they simultaneously regulate pro-apoptosis, anti-apoptosis, and proliferation processes; hence, every single cell maintains its own-JNK activity-based balance between cell death and survival^34,35^. Therefore, JNKs are considered promising therapeutic candidates since they can be targeted to modulate many factors, and cells show different results in response to the same stimulation^34,36,37^.

Unlike in non-cancerous cells, the level of JNK activation is considerably high in cancerous cells, such as cells of hepatocellular carcinomas (HCCs) and human pancreatic cancer cells, because JNK activity promotes tumor growth, proliferation, and survival^61–63^. Furthermore, to counteract the effects of anti-tumor drugs or damage (e.g., ER stress, microbial infection, and accumulation of protein aggregates) tumor cells require JNK cascades to promote autophagy^57,64–66^. In addition, JNK activity plays a critical role in mechanisms of immune evasion to protect cancerous cells from immune surveillance and subsequent destruction^67,68^. In this study, the viability of cancerous HepG2 cells decreased at low concentrations of EEDK, suggesting that even that amount is sufficient to exceed the normal range of JNK activation and disrupt the balance between cell survival and death that results in JNK-AP-1/p53 mediated apoptosis. However, we found that normal JNK activation in non-cancerous HEK293A cells was relatively low and that low concentrations of EEDK (0.1–1 μg/mL) were not harmful and did not lead to the over-activation of JNKs. Instead, it was helpful for cell proliferation. In HEK293A cells treated with 50 μg/mL EEDK, a concentration that was detrimental for HepG2 cells, we observed that HEK293A cells were still able to form colonies after change of growth medium indicating that HEK293A cells were able to endure and adapt to the injury induced by increased JNK activation. Second, according to a previous study, EEDK is composed of various flavonoids, such as quercetin and kaempferol, and their glycoside, galactoside, or galloylated derivatives^13^.

Interestingly, quercetin and kaempferol, which are major components of EEDK, play diametrically opposing roles in the activation and inhibition of JNK phosphorylation, respectively^69–71^. The approximate ratio of quercetin and kaempferol, including the derivatives of each, in EEDK is 3:5^13^. Moreover, a high concentration of EEDK did not induce abnormal contraction or shrinkage of cells, and treatment with various drugs showed predictable results, thereby suggesting that the biologically friendly compounds in EEDK can preserve the cells appropriately, with normal function. We suggest that these specific properties of EEDK could lead to cell survival or death according to cell type. Nevertheless, these results require further investigation. It would be interesting to modify the ratio of quercetin and kaempferol and study the normal range of JNK activity in each cell type and use this knowledge to develop drugs that target specific cells.

Although data from the Fluo-3 assay showed that EEDK treatment caused an increase in intracellular Ca^2+^ levels, we found that EEDK treatment also decreased NFAT activity. Calcineurin, which is activated by Ca^2+^, has previously been shown to dephosphorylate and activate NFAT^46,47^. Hence, there is a discrepancy between our results and previously published data. According to Dolmetsch et al., a massive transient influx of calcium could activate other signaling intermediates, such as JNKs and NF-κB and, therefore, may not lead to sustained nuclear translocation and activation of NFAT proteins^72^. Moreover, EEDK-treated cells mainly followed the JNK signaling pathway, which can phosphorylate and inactivate NFAT^48,49^.

By using FRET live imaging and genetically-encoded biosensors, we first visualized the EEDK-induced PDGFR activation. The significant components of the Kras-PDGFR biosensor are the substrate sequence containing Tyr751 of PDGFRβ and the Src homology 2(SH2) domain of Nck2, an adaptor protein that associates with the tyrosine-phosphorylated growth factor receptor^73^. When the Tyr751 site is phosphorylated, the SH2 domain binds to the substrate, resulting in a conformational change in the biosensor and a change in the FRET ratio. In the current study, we found that the FRET ratio change of the Kras-PDGFR biosensor occurred in response to EEDK, suggesting that diverse variables induced the phosphorylation of Tyr751. It is intriguing that the SH2 domain of Nck2 rapidly bonds to p-Tyr751 after just 4 min. In the previous study, the Nck2 bound to PDGFR interacts with Nck interacting kinase (NIK) via the SH3 domain; the correlation could modulate MEKK1 activity and result in JNK/SAPK pathway^74^. According to our results and previous reports, we founded that EEDK activated the JNK pathway through various routes.

**Figure 8:**
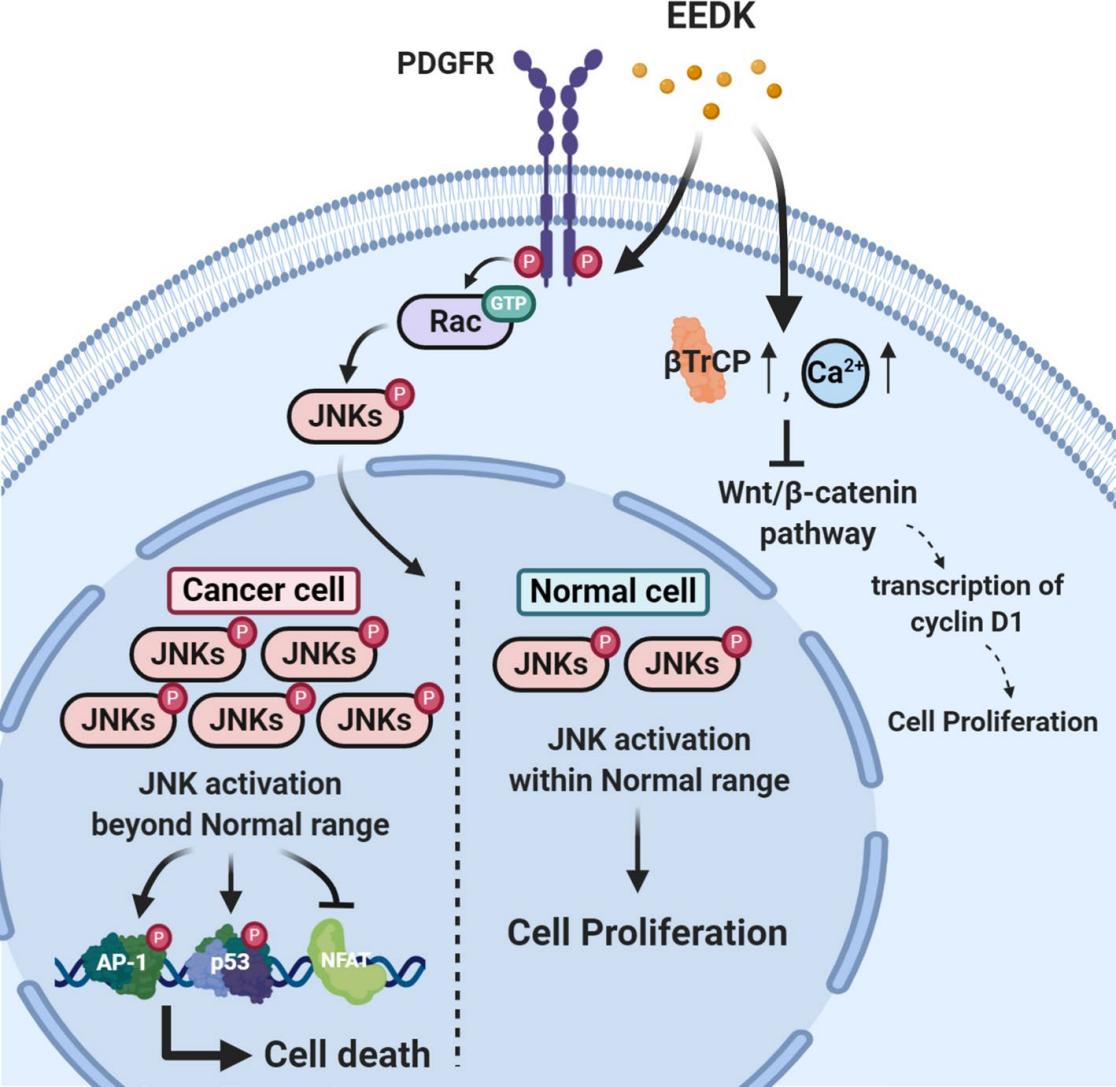
A proposed model of the EEDK-induced cell death pathway. EEDK could activate the PDGFR-Rac-JNK signaling axis. In cancerous cells, which have high basal JNK activity, enhanced JNK activity exceeds a normal range of JNK activation, leading to JNK-AP-1/p53 mediated cell death. However, the same amount of EEDK is not sufficient to exceed the normal range of JNK activity in normal cells, which has low basal JNK activation, resulting in cell proliferation. Besides, EEDK could suppress Wnt/β-catenin pathway by promoting βTrCP expression and intracellular Ca^2+^ concentration. This figure was created using BioRender (https://biorender.com/).

In conclusion, we observed that specific concentrations of EEDK induce cellular death and are more sensitive to cancerous cells than non-cancerous cells. FRET live imaging using biosensors allowed us to show that EEDK treatment may affect cells via the PDGFR-Rac-JNKs pathway. Additionally, using several drugs, we confirmed that the JNKs and their downstream transcription factors (e.g., AP-1 and p53) were involved in EEDK-induced cell death (Fig. 8). These data provide novel insights into natural products that are composed of various flavonoids, and we propose that EEDK may be a promising anti-tumor agent. As far as we know, EEDK has not yet been applied to a tumor in vivo model. Follow-up studies will, therefore, need to examine the appropriate amount of EEDK working on tumors or in the clinical trial. Furthermore, metabolic analysis of samples will help to determine which components of EEDK cause these therapeutic effects.

## Methods

### Gene construction and DNA plasmid cloning

The βTrCP-EYFP was constructed by restriction enzyme cloning. Using p4489 Flag-βTrCP (Cat no. 10865, Addgene, Watertown, MA), βTrCP was amplified by PCR and ligated into pEYFP-C1 (TaKaRa Bio USA, Inc., Mountain View, CA) via the AgeI and NheI sites. The primers used in the PCR are as follows: forward 5′-TAATGCTAGCGCCACCATGGACTAC-3′ and reverse 5′-GAGCACCGGTCTTCTGGAGATGTAGGT-3′. The Kras-PDGFR biosensor was kindly provided by Dr. Jihye Seong (Korea Institute of Science and Technology, Republic of Korea). The Rac biosensor was a gift from Dr. Yingxiao Wang (University of California, San Diego, CA). JNKAR1EV-NLS was also constructed by restriction enzyme cloning. The DNA sequence of the Ypet-FHA1-EV linker-JNK substrate was excised from the hyBRET-JNK-EV construct (Cat no. 108656, Addgene) using EcoRI and NotI restriction enzymes. After 4048NLS, which contains the Ypet-ECFP FRET pair and the NLS, was excised using the same enzymes, the NLS-containing vector and insert containing the JNK-specific substrate were ligated. Dr. Michiyuki Matsuda (Kyoto University, Japan) kindly gifted the 4048NLS. Additionally, 3xAP1pGL3 (Cat no. 40302, Addgene) and pGL3-NFAT luciferase (Cat no. 17870, Addgene) were kind gifts from Alexander Dent and Jetty Crabtree, respectively.

### Cell culture and transfections

HEK293A and HepG2 cells were cultured in Dulbecco’s modified Eagle’s medium (DMEM; CM002, GenDEPOT, Katy, TX) supplemented with 10% (v/v) fetal bovine serum (FBS; WB0015, HyClone, Logan, UT), 100 U/mL penicillin, and 100 μg/mL streptomycin (CA005, GenDEPOT, Katy, TX). The cells were cultured in a humidified incubator with 95% air and 5% CO_2_ at 37 °C. The DNA plasmids were transfected into the cells using Lipofectamine 3000 (Invitrogen, Carlsbad, CA), according to the manufacturer’s instruction.

### Plant materials and chemicals

The protocol for ethanol extraction of *D. kaki* was well described in our previous study^13^. EEDK was dissolved in Dimethyl sulfoxide (DMSO; Biosesang, Seongnam, Republic of Korea) prior to cell treatments. DMSO was used as a negative control, and the final DMSO concentration did not exceed 0.5% (v/v). Fluo-3 was purchased from Invitrogen (Carlsbad, CA). Epidermal growth factor (EGF) and cobalt chloride (CoCl_2_) were purchased from Sigma (St. Louis, MO). T-5224 and anisomycin are commercially available from MedChemExpress (Monmouth Junction, NJ). Pepstatin A was obtained from ENZO Life Science (Farmingdale, NY).

### Viability assays

The WST-8 assay was used to determine cell viability. HEK293A cells and HepG2 cells were seeded at 8 × 10^3^ cells/well in 96-well plates and incubated for 24 h at 37 °C before cells were treated with a DMEM-containing control (0.5% DMSO) or EEDK (0.1–100 μg/mL), with or without different drugs for 24 h. After washing, the cells were treated with 9.09% (v/v) Cellrix Viability Assay kit (B1007-500, MediFab, Seoul, Republic of Korea) in DMEM without phenol red (Cat no. 31053028, Gibco, Waltham, MA) for 2 h at 37 °C. The optical density of the solubilized formazan product was measured using a Glomax Multi + Microplate Multi Reader (9301–010, Promega, USA) at a wavelength of 450 nm.

### Clonogenic assay

Cells were seeded at 1 × 10^3^ cells/plate in 70 mm cell and tissue culture dishes (TCD010070, Biofil, China) and incubated for 24 h at 37 °C. After the medium was removed, cells were treated with DMEM containing 0.5% (v/v) DMSO or different concentrations of EEDK (50 μg/mL or 100 μg/mL) for 30 h at 37 °C. Next, the medium was replaced with growth medium (DMEM with 10% (v/v) FBS), and HEK293A and HepG2 cells were incubated for 9 and 6 days, respectively. Cells were washed before they were fixed in 4% (v/v) paraformaldehyde (P2031, Biosesang) for 10 min. After washing, cells were incubated with 0.5% (v/v) crystal violet (C0775, Sigma) in 25% (w/v) methanol for 15 min at room temperature. Cell colonies were quantified using ImageJ software^75^ version 1.52 (National Institutes of Health, Bethesda, MD; https://imagej.nih.gov/ij/) .

### Fluorescence intensity detection

Fluo-3 assays were used to study intracellular calcium concentrations. HEK293A and HepG2 cells were seeded at 1 × 10^4^ cells/well in 96-well plates and incubated for 24 h at 37 °C. After a 24 h starvation with DMEM containing 0.5% (v/v) FBS, cells were exposed to medium containing 0.5% (v/v) DMSO or EEDK (50 μg/mL) for 8 h. Cells were washed before they were treated with 4 μM Fluo-3 in DMEM for 45 min at 37 °C. After washing, cells were incubated in DMEM without phenol red, and the fluorescence intensity was analyzed. To evaluate βTrCP expression, the fluorescence intensity of βTrCP-EYFP in the transfected cells was measured. HEK293A cells and HepG2 cells were seeded at 1 × 10^4^ cells/well in 96-well plates and incubated for 24 h at 37 °C before βTrCP-EYFP transfection. After being starved with DMEM supplemented with 0.5% (v/v) FBS for 24 h, DMEM containing control (0.5% (v/v) DMSO) or EEDK (50 μg/mL) was used to treat cells for 24 h. Next, cells were washed, and the medium was replaced with DMEM without any phenol red, and the fluorescence intensity was analyzed. The fluorescence intensities of the Fluo-3—Ca^2+^ complex and βTrCP-EYFP were measured using a Glomax Multi + Microplate Multi Reader (9301-010, Promega, USA) at 490 nm excitation and 510–570 nm emission.

### Image acquisition and microscopy

Cells expressing several exogenous proteins were cultured in a confocal dish (Cat no. 100350, SPL Life Sciences, Republic of Korea) and starved using DMEM containing 0.5% (v/v) FBS for 24 h before imaging. Shortly before the experiment, cells were washed, and the medium was replaced with CO_2_-independent medium (Cat no. 18045088, Gibco, Waltham, MA) containing 0.5% (v/v) FBS and 4 mM L-glutamine. Images were obtained on a Leica DMi8 microscope equipped with a charge-coupled device (CCD) camera (DFC450C, Leica, Germany) and a 436/20 excitation filter, a 455 dichroic mirror, and two emission filters controlled by a filter changer (480/40 for ECFP and 535/30 for YPet). LASX software version 3.6.0. (Leica, Germany; https://www.leica-microsystems.com/products/microscope-software/p/leica-las-x-ls/) was used to acquire images and compute the emission intensity of ECFP and FRET. A specific region of target cells was selected as a region of interest (ROI) to observe signals and perform quantification. The fluorescence intensity in the background region was selected and quantified to take the signal away from the ROI of ECFP and FRET channels. Quantified values were analyzed using GraphPad Prism version 7.0.0 for Windows (GraphPad Software, La Jolla, CA; https://www.graphpad.com/).

### Luciferase assay

Cells were seeded at 1 × 10^4^ cells/well in 96-well plates and incubated for 24 h at 37 °C. Next, 3xAP1pGL3 and pGL3-NFAT luciferase constructs were transfected into cells using Lipofectamine 3000 (Invitrogen, Carlsbad, CA). After incubation for 24 h and washing, cells were starved with DMEM supplemented with 0.5% (v/v) FBS for 24 h before drug treatment. Transfected cells were treated with the control (DMEM and 0.5% (v/v) DMSO) or EEDK (50 μg/mL) for 24 h. Cells were then harvested in 1X cell lysis buffer, and luciferase activity was measured using the Pierce Firefly Luciferase Glow assay Kit (16176, Thermo Fisher Scientific, Waltham, MA), according to the manufacturer’s instructions. The optical luminescence of the luciferase was measured using a Glomax Multi + Microplate Multi Reader (9301-010, Promega, USA).

### Statistical analysis

All results are expressed as the mean ± standard error of the mean (S.E.M). Statistical analyses were performed using the unpaired Student’s *t*-test to determine the statistical significance of difference between the two mean values. We considered a *P*-value < 0.05 to be statistically significant.
